# Perforated gastric corpus in a strangulated paraesophageal hernia: a case report

**DOI:** 10.1186/1752-1947-3-6507

**Published:** 2009-05-07

**Authors:** Alexis E Shafii, Steven C Agle, Emmanuel E Zervos

**Affiliations:** 1 Department of Thoracic and Cardiovascular Surgery, The Cleveland Clinic, 9500 Eucid Avenue, Cleveland, Ohio 44195, United States; 2 Department of Surgery, East Carolina University, 600 Moye Boulevard, Greenville, North Carolina 27834, United States

## Abstract

**Introduction:**

Patients with paraesophageal hernias often present secondary to chronic symptomatology. Infrequently, acute intestinal ischemia and perforation can occur as a consequence of paraesophageal hernias with potentially dire consequences.

**Case presentation:**

An 86-year-old obtunded male presented to the emergency department with hypotension and severe back and abdominal pain. An emergency abdominal CT scan was ordered with a presumptive diagnosis of ruptured abdominal aortic aneurysm. CT topograms revealed extensive free intra-abdominal air and herniated abdominal viscera into the right hemithorax. Prior to completion of the CT study, the patient sustained a cardiopulmonary arrest. Surgery was consulted, but the patient was unable to be revived. Post-mortem examination revealed gross contamination within the abdomen and a giant, incarcerated, hiatal hernia with organoaxial volvulus and ischemic perforation.

**Conclusion:**

Current recommendations call for prompt repair of giant hiatal hernias before they become symptomatic due to the increased risk of strangulation. Torsion of the stomach in large hiatal hernias frequently leads to a fatal complication such as this warranting elective repair as soon as possible.

## Introduction

Paraesophageal hernias occur when intra-abdominal contents herniate through the esophageal hiatus into the mediastinum. There are four types: type I occurs when the stomach slides into the mediastinum thus displacing the gastroesophageal junction into the thorax (sliding hiatal hernia), type II and III paraesophageal hernias result from herniation of the stomach through the esophageal hiatus and subsequent organoaxial and mesoaxial rotation respectively, and type IV hernias involve organs other than the stomach herniating through the hiatus into the thorax. Current recommendations are for prompt repair secondary to the possibility of complications including hemorrhage, ischemia, and perforation [1].

## Case Presentation

**Figure 1:**
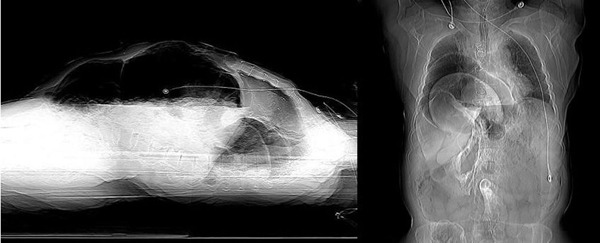
**Intraabdominal free air seen on lateral abdominal topogram and herniated abdominal viscera on supine view**.

**Figure 2:**
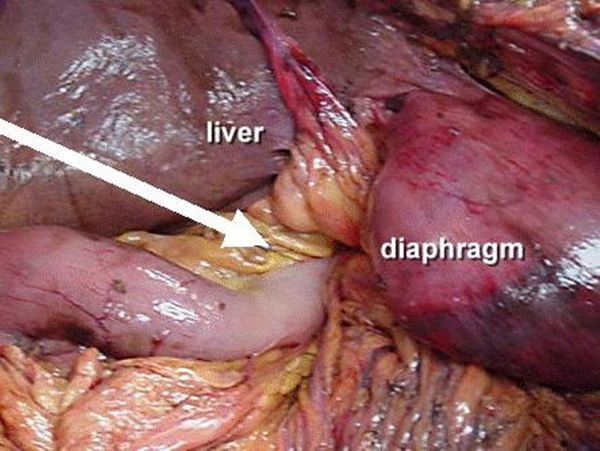
**Pointer on pylorus at esophageal hiatus**.

**Figure 3:**
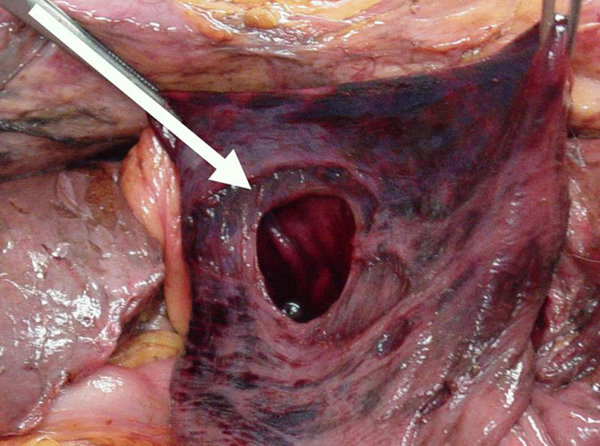
**Perforation of gastric fundus**.

An 86-year-old white, American, male presented to the emergency department hypotensive and obtunded with severe abdominal and back pain of unknown duration. A ruptured abdominal aortic aneurysm was initially suspected and the patient was taken for an abdominal CT scan at the request of the emergency room physicians. After completion of a thoracoabdominal topogram, a large quantity of free intra-abdominal air was seen on the lateral view and herniated abdominal viscera were identified in the right chest on the supine view (Figure 1). Prior to completion of the scan, the patient succumbed to a cardiopulmonary arrest. A postmortem examination of the abdomen and chest was performed. Upon entering the abdominal cavity there was gross contamination as well as a giant incarcerated hiatal hernia. The entire stomach was herniated up and into the posterior mediastinum with only the pylorus visible at the hiatus (Figure 2). Remnants of a failed hiatal hernia repair were found along the diaphragmatic extension of the left crus. Once the stomach was freed from adhesions and reduced into the abdominal cavity the site of an ischemic perforation of the gastric fundus was identified (Figure 3).

The patient's past medical history was uncovered post-mortem and was significant for a prior coronary artery bypass, congestive heart failure, and previous hiatal hernia repair. The initial discovery of a giant hiatal hernia was made thirteen years prior by esophagogastroduodenoscopy during an evaluation for coffee ground emesis and chronic anemia. Repair was performed at that time and consisted of primary crural re-approximation and gastropexy. Late recurrence of the giant hiatal hernia was also documented but re-operation was not undertaken due to his poor cardiac reserve.

## Conclusion

Torsion of the stomach in these very large hiatal hernias can lead to fatal complications with considerable frequency, and as a result, elective repair is warranted upon discovery except in the moribund patient [2]. Emergent surgical intervention in the case of a complete gastric volvulus involves reduction of the volvulus and hiatal repair [3]. Patients with this condition often present with a classic triad composed of retching, epigastric pain, and failure to place a nasogastric tube. Partial gastrectomy may also be required in cases of infarcted stomach or perforation. Optimal elective repair involves reduction of the hernia, excision of the hernia sac, and repair of the hiatal defect, which if excessively large, may require prosthetic mesh reinforcement [4]. Collis-Nissen fundoplication may be added to the repair to accommodate relative esophageal shortening but not without risk of dysmotility of the distal esophagus [5]. While traditionally these repairs were approached via celiotomy or thoracotomy, the majority of cases are now amenable to laparoscopic approaches with excellent outcomes [6]. Indeed, in the referenced study, 200 consecutive patients underwent laparoscopic repair of paraesophageal hernias with only one death, low morbidity, and a 2.5% recurrence rate.

It is evident that this patient's pathology was the consequence of a chronically incarcerated giant hiatal hernia left untreated, which ultimately led to his demise. While it remains unclear as to what his true surgical risks were, we currently recommend that most patients can be repaired with low morbidity and nearly zero mortality.

## List of abbreviations

CT: Computerized tomography.

## Consent

Written informed consent has been attained from the deceased patient's family to publish information related to the case as well as images associated with the case.

## Competing interests

The authors declare that they have no competing interests'.

## Authors' contribution

AS and EZ were both involved in the conception and data gathering for the case report. SA was involved in drafting and revising the manuscript. AS, EZ and SA were involved in the literature review and obtaining the critical intellectual content used in this case report. These three authors have also given final approval for publication.
